# Supplementary material for: Implicit reward-based motor learning
Source: Exp Brain Res. 2023 Aug 14;241(9):2287–98. doi: 10.1007/s00221-023-06683-w (PMC10471724; doi:10.1007/s00221-023-06683-w)
Supplement: Supplementary file 2 — Supplementary file2 (PDF 171 kb) [file 221_2023_6683_MOESM2_ESM.pdf]

Implicit reward-based motor learning

N.M. van Mastrigt[1], J.S. Tsay[2], T. Wang[2], G. Avraham[2], S.J. Abram[2], K. van der Kooij[1], J.B.J. Smeets[1] & R.B. Ivry[2]

[1] Vrije Universiteit Amsterdam, Department of Human Movement Sciences, Amsterdam, The Netherlands
[2] UC Berkeley, CognAc lab, Berkeley, California, United States

Corresponding author: N.M. van Mastrigt, n.m.van.mastrigt@vu.nl

---

Experimental Brain Research
Supplementary information

Online resource 2 – Learners and non-learners

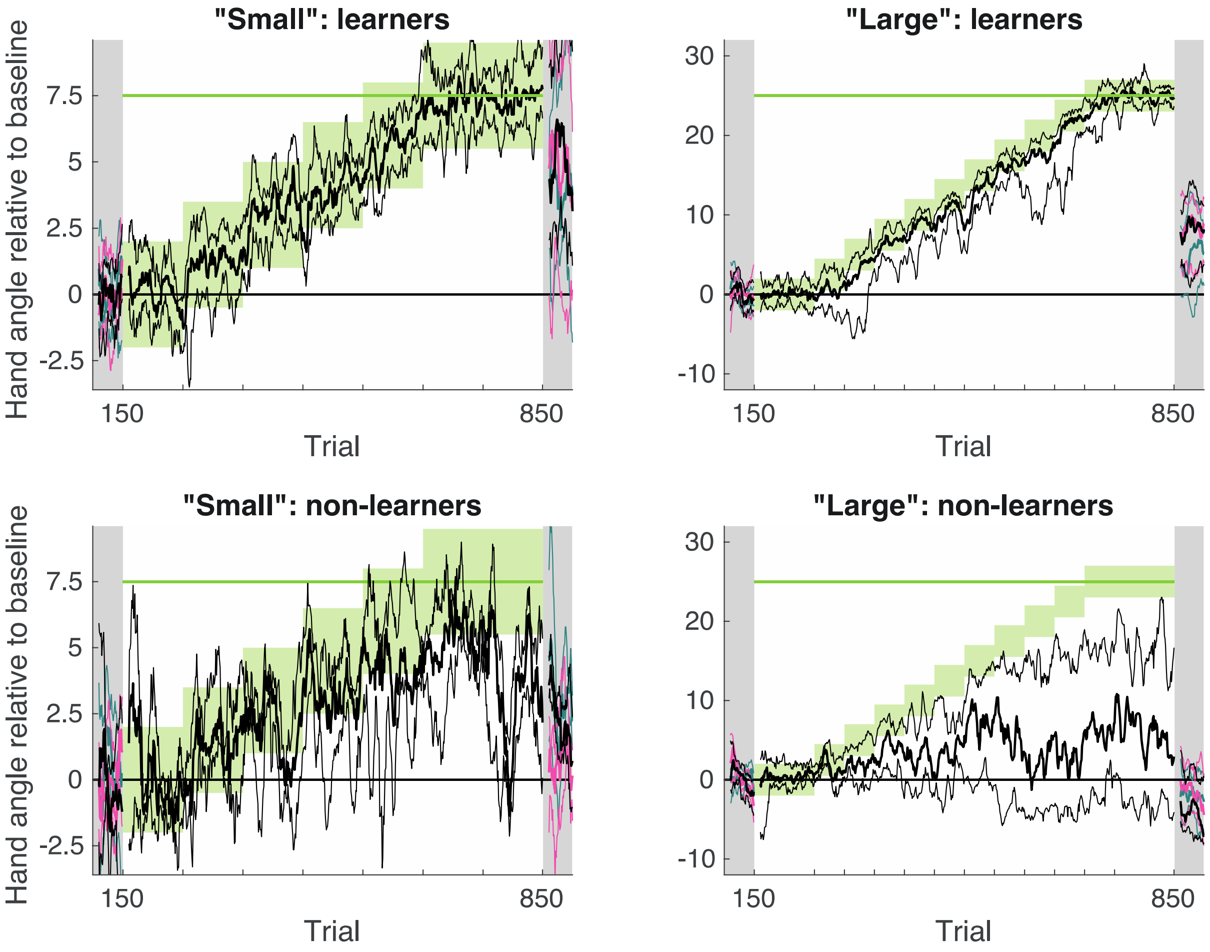

Online Resource 2. Learners and non-learners. Participants were divided into two groups based on whether final learning was in the reward zone ("learners", top rows) or not ("non-learners", bottom rows). By this definition, there were 16 learners and 4 non-learners in the Small Perturbation group (left) and 16 learners and 12 non-learners in the Large Perturbation group.
