# Supplementary material for: Implicit reward-based motor learning
Source: Exp Brain Res. 2023 Aug 14;241(9):2287–98. doi: 10.1007/s00221-023-06683-w (PMC10471724; doi:10.1007/s00221-023-06683-w)
Supplement: Supplementary file 3 — Supplementary file3 (PDF 314 kb) [file 221_2023_6683_MOESM3_ESM.pdf]

Implicit reward-based motor learning

N.M. van Mastrigt[1], J.S. Tsay[2], T. Wang[2], G. Avraham[2], S.J. Abram[2], K. van der Kooij[1], J.B.J. Smeets[1] & R.B. Ivry[2]

[1] Vrije Universiteit Amsterdam, Department of Human Movement Sciences, Amsterdam, The Netherlands
[2] UC Berkeley, CognAc lab, Berkeley, California, United States

Corresponding author: N.M. van Mastrigt, n.m.van.mastrigt@vu.nl

---

Experimental Brain Research
Supplementary information

Online resource 3 – Post-experiment questionnaire results

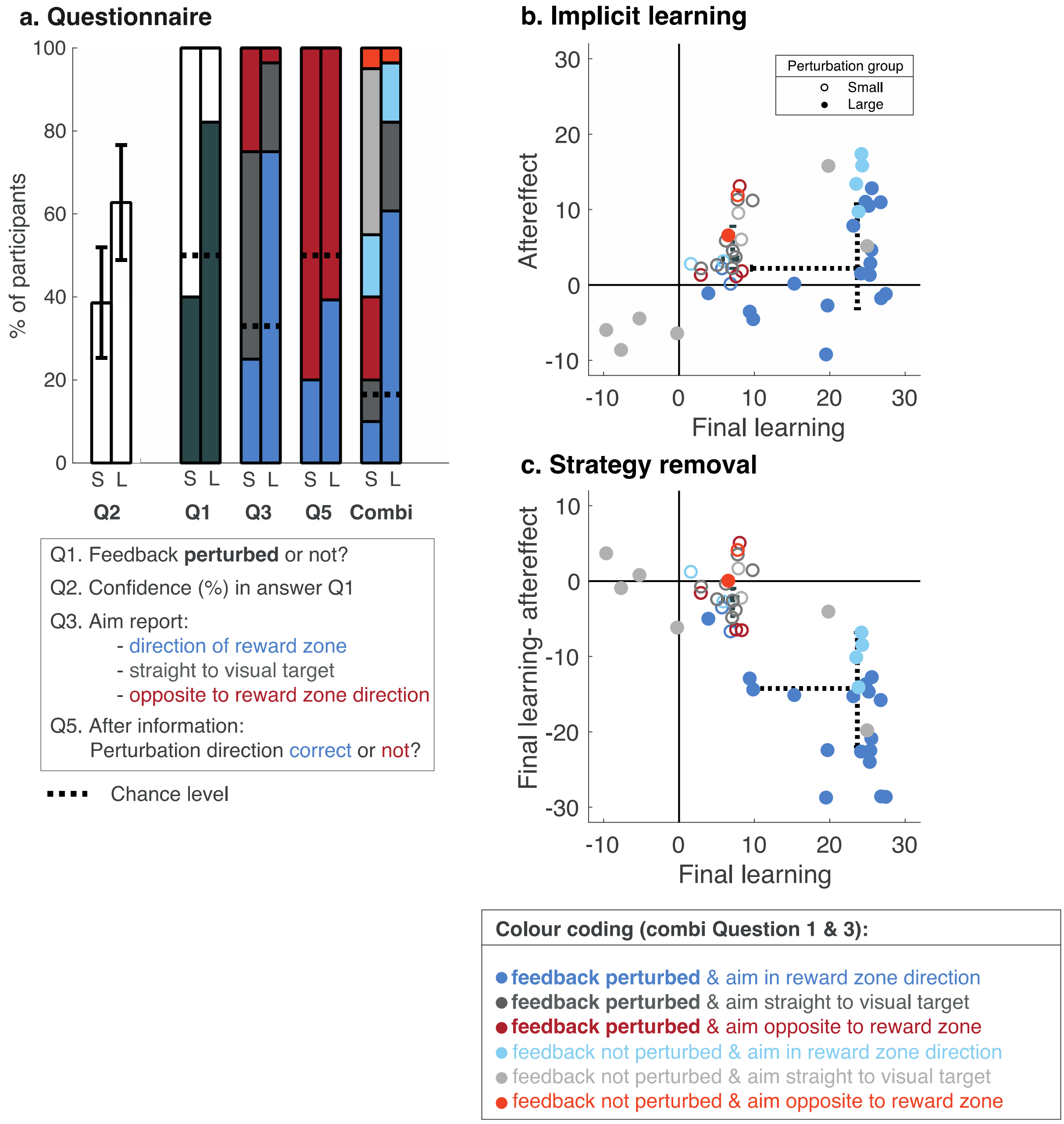

Online Resource 3. a. Questionnaire results related to learning for the Small Perturbation group (left bars) and the Large Perturbation group (right bars). Q's correspond to question numbers on the questionnaire. See Online Resource 1 for the post-experiment questionnaires for the Small perturbation group and Large perturbation group. Q4 is missing as it was used to exclude participants who reported to have aimed off-target during the aftereffect phase. b. Implicit learning, color-coded for responses to question 1 and 3 on the questionnaire. c. Strategy removal, color-coded for responses to question 1 and 3 on the questionnaire. Note that the large variability, especially in the aftereffect measure for those who reported using a strategy (blue symbols).
