# Supplementary material for: Implicit reward-based motor learning
Source: Exp Brain Res. 2023 Aug 14;241(9):2287–98. doi: 10.1007/s00221-023-06683-w (PMC10471724; doi:10.1007/s00221-023-06683-w)
Supplement: Supplementary file 4 — Supplementary file4 (PDF 161 kb) [file 221_2023_6683_MOESM4_ESM.pdf]

Implicit reward-based motor learning

N.M. van Mastrigt[1], J.S. Tsay[2], T. Wang[2], G. Avraham[2], S.J. Abram[2], K. van der Kooij[1], J.B.J. Smeets[1] & R.B. Ivry[2]

[1] Vrije Universiteit Amsterdam, Department of Human Movement Sciences, Amsterdam, The Netherlands
[2] UC Berkeley, CognAc lab, Berkeley, California, United States

Corresponding author: N.M. van Mastrigt, n.m.van.mastrigt@vu.nl

---

Experimental Brain Research
Supplementary information

Online resource 4 – Reach angle variability

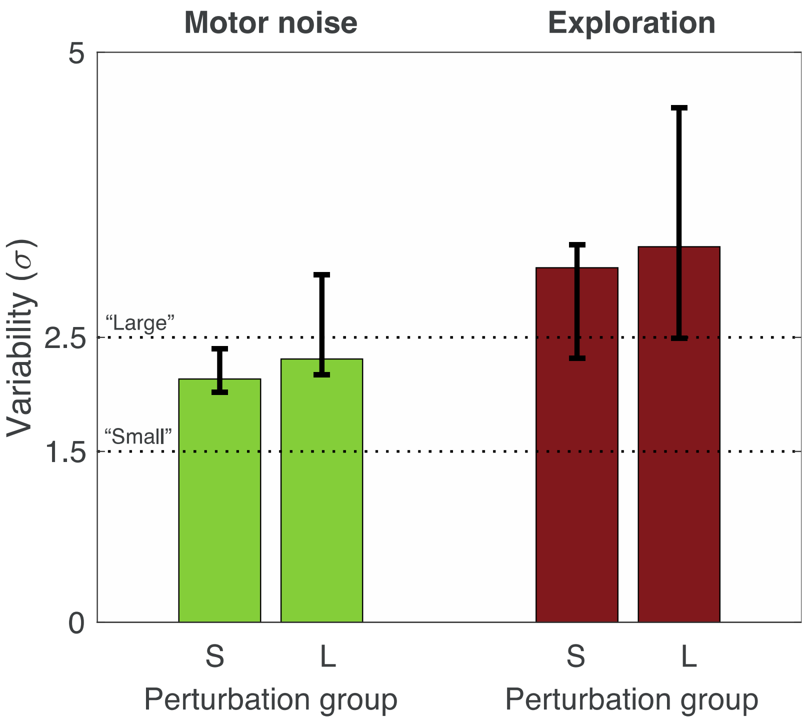

Online Resource 4. Reach angle variability separated into motor noise and exploration. Motor noise can be considered inevitable variability following rewarded trials, whereas exploration can be considered additional variability following non-rewarded trials. These estimates are based on the ATTC-method with the simplest reward-based motor learning model (van Mastrigt et al., 2021)*. Median and interquartile range over participants for the Small perturbation group (S) and Large perturbation group (L). Horizontal dotted lines indicate step sizes of the gradual perturbation.*

References

van Mastrigt, N. M., van der Kooij, K., & Smeets, J. B. J. (2021). Pitfalls in quantifying exploration in reward-based motor learning and how to avoid them. *Biological Cybernetics*, *115*(4), 365–382. https://doi.org/10.1007/s00422-021-00884-8
